# Tissue requirements for the application of aortic valve neocuspidization – appropriate pericardium properties and homogeneity?

**DOI:** 10.1007/s10856-024-06790-2

**Published:** 2024-04-29

**Authors:** Claudia Dittfeld, Sophia Bähring, Cindy Welzel, Anett Jannasch, Klaus Matschke, Sems-Malte Tugtekin, Konstantin Alexiou

**Affiliations:** Department of Cardiac Surgery, Carl Gustav Carus Faculty of Medicine, Technische Universität Dresden, Heart Centre Dresden, Dresden, Germany

## Abstract

Objective: Aortic valve neocuspidization (AVNeo) using autologous pericardium is a promising technique. Expected advantages are reduced immune response, appropriate biomechanics and lower treatment expenses. Nevertheless, autologous pericardium can be affected by patient’s age and comorbidities. Usually, glutaraldehyde (GA) - fixed bovine pericardium is the basic material for aortic valve prostheses, easy available and carefully pre-examined in a standardized fabrication process. Aim of the study is the verification of autologous pericardial tissue homogeneity by analysing tissue thickness, biomechanics and extracellular matrix (ECM) composition. Methods: Segments of human GA-fixed pericardium selected by the surgeon based on visual criteria for cusp pre-cut and remaining after surgical AV replacement were investigated in comparison to bovine standard tissue treated equivalently. Pericardium sampling was performed at up to three positions of each sutured cusp for histological or biomechanical analysis, according to tissue availability. Results and Conclusions: Human pericardia exhibited a higher heterogeneity in collagen content, density of vessel structures and elastic moduli. Thickness, vessel density and collagen and elastin content differed significantly between the species. In contrast, significant interindividual differences were detected in most properties investigated for human pericardial samples but only for tissue thickness in bovine tissues. Higher heterogeneity of human pericardium, differing vessel and collagen content compared to bovine state-of-the-art material might be detrimental for long term AV functionality or deterioration and have to be intensely investigated in patients follow up after autologous cusp replacement.

**Graphical Abstract:**

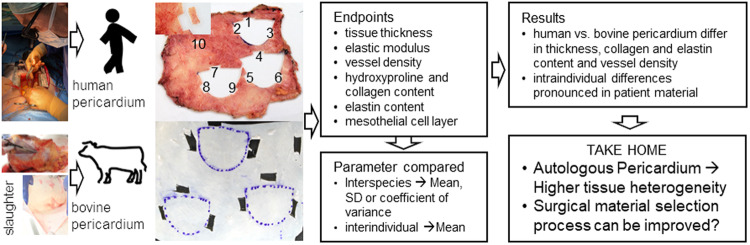

## Introduction

Already since decades autologous human pericardia are used for AV replacement, named Aortic Valve Neocuspidization (AVNeo) but in literature also Ozaki procedure according to the recent establishment by Shigeyuki Ozaki [1–7]. Prior to autologous cusp implantation patient’s pericardium is excised in cardiac surgery, treated with 0.6% glutaraldehyde solution for ten minutes and rinsed three times in physiologic saline solution [3, 8]. Favorable midterm outcomes are proven after a period of up to 10 years in a patient cohort of 481 [3]. Clinical studies confirm excellent survival, a low rate of reoperation at middle-term follow-up, and good hemodynamic improvement also if outcome after bioprostheses implantation was compared [5, 8–11]. In particular the physiological movement of the aortic annulus through the cardiac cycle after AVNeo has to be highlighted [5, 12]. Due to the 10–15-year durability of conventional bioprostheses fabricated from bovine pericardium, especially for younger patients that have the risk for necessary re-operation, the use of autologous pericardium is a promising alternative [2, 13]. A discussed increase of free margin lengths of the cusps in AVNeo can be the basis for a greater utility even for pediatric populations with continued aortic annular growth [1, 14, 15]. But also adult or elderly patients with an increased risk of prosthesis-patient mismatch e. g. with extreme anatomical specificities may profit from the surgical technique using autologous material [5, 13]. Nevertheless, in 2022 a first case with the need for re-operation after AVNeo was published describing the calcification of the cusps at the annulus positions [16].

Most important advantage of the autologous pericardium is the reduced immunogenicity which is described to be induced mainly by xeno-epitopes such as the carbohydrate moiety galactosyl-α-1,3-galactose (α-Gal) or Neu5Gc (glycolyl form of neuraminic acid) after implantation of bovine or porcine pericardium despite GA-fixation [17–19]. Independent from these xenogeneic epitopes and investigating autologous implantation in rabbit animal models a reduced calcification potential was published for the autologous tissue [20]. A detailed in vitro and microbiological analysis of human pericardia was presented by Straka et al. comparing the tissues with original human AV tissues, leading to the conclusion that human pericardia exhibit advantageous ECM biopolymeric structure and biomechanical properties [18]. Gardin et al. investigated human pericardia pre and post GA-fixation process derived from surgical procedure material [21]. Native human tissues showed an intact mesothelial cell layer and typical collagen bundle morphology. After GA-fixation process the mesothelial layer was partially destroyed and underlying collagen matrix appeared more densely packed and more compact. Endothelial cell repopulation was comparable to bovine patch material [21]. These laboratory results in addition to the promising clinical midterm outcome confirm the suitability of the autologous pericardium for AV replacement. Nevertheless, patient’s materials depend on comorbidities and age of the individual. In contrast to quality assured bovine pericardium in industrial fabrication, human tissues are not pre-evaluated prior to cusp excision in the theater. This can be achieved by using bovine pericardial patch material instead of autologous tissue on the one hand applying the Ozaki-protocol but also by preparation of a mono-patch pre-cut according to the Batista et al. established already in the eighties [22–25]. Clinical outcomes using bovine pericardium without stent in the Ozaki-like procedure have recently been turned out to even result in inferior results and decreased operation times (cardiopulmonary bypass time 143.8 min in AVNeo and 90.3 min in surgery using bovine pericardium) due to the possibility to prepare the patch before the surgical procedure starts [24, 25]. Mortality after AVNeo using human pericardium was with 2.7% higher and survival rate of 85.9% lower than in surgery using bovine pericardium patch (1.2% mortality and 94.7% survival rate) [25]. Nevertheless immunological disadvantages by using xenogeneic material can essentially impact the longevity of aortic valve replacement tissue and have even more effect than tissue properties. Aim of the presented study was to determine the heterogeneity of human pericardium used for AV replacement in cardiac surgery adjacent to the excised AV cusps. Pericardium thickness, biomechanics, hydroxyproline content are evaluated and ECM composition, vessel density and mesothelial cell layer are proven from histological staining in comparison to bovine standard pericardium treated equivalently. Bovine tissues spanning the right ventricle and corresponding regions “C-E” and in part region “M” described by Stiegelmeier et al. were used in the presented setup [26], as respective human pericardia are accessible in theater. Increased heterogeneity of human pericardia and individual suitability of patient tissues might be relevant for long term stability in the extreme biomechanical AV position. Decision making process for the right area might include measurement of tissue thickness after GA-fixation with low efforts in the operating theater. But also the spontaneous switch to bovine pericardial patches or bioprostheses should be considered, if autologous tissue appears inappropriate.

## Patients, materials and methods

### Pericardial tissues

Human pericardia (*n* = 6) were obtained after excision of three cusps for AV reconstruction. Written informed consent form was signed by patients. The study was approved by the ethics committee of the Dresden University (Ethikkommission an der TU Dresden, registration number EK 247052019). All patients whose pericardium was implemented in the study were male and between 55 and 75 (66.5 ± 7.5) years old. BMI was between 26 and 31.9 (29.6 ± 2.2). Five of the patients suffered from aortic valve stenosis, one patient from aortic valve insufficiency. Five of the patients took lipid-lowering drugs but only two of them antidiabetics. Patients with previous cardiac surgery or history of pericarditis. All patients received isolated aortic valve replacement.

In theater pericardia were extracted and fixed in 0.6% GA-solution in buffer for ten minutes as described by Ozaki et al. [3]. Material is rinsed three times in physiologic saline solution prior cusp selection. Remaining pericardia were immediately transported to laboratory and sampling was performed adjacent to the excised cusps areas at three margins, if available.

Bovine pericardia (*n* = 3) from female cattle of the race Salers were obtained from a local slaughterhouse (Vorwerk Podemus ökologischer Landbau, Dresden, Germany). Two animals were 16 months old, the third animal was six years and eight months old. After removal of surrounding fatty and connective tissues, the pericardium was washed several times with phosphate-buffered saline (PBS). Apes to base orientation was considered during preparation. According to the AVNeo-procedure where the human pericardia that cover the right ventricle of the heart are used, the respective area of the bovine pericardial sack was prepared. This corresponds to the areas “C to E” and partially “M” described in Stiegelmeier et al. [26]. GA-fixation (0.6%) step was extended to 20 min interval due to the higher tissue thickness.

### Biomechanics

Pericardium was cut into rectangles of 10 × 15 mm and tissue thickness was determined using thickness gauge FD50 (Kaefer Messuhrenfabrik GmbH & Co. KG, Villingen-Schwenningen, Germany). Uniaxial tensile testing was performed with the Minimat Miniature Materials Tester (Polymer Lab., Dresden, Germany) clamping both ends of the sample into the wedges of the instrument. Clamping lengths of 7.0–8.5 mm were applied due to adjustment of a 0.02 N pre-load. Tensile testing was performed with a deformation rate of 4 mm/min and stopped at a tensile force of 10 N or an elongation of 10 mm. After plotting the stress-strain curves the Young’s modulus (E) was defined as the slope of the linear function of the respective part of the stress-strain curve.

### Hydroxyproline assay

To quantify collagen content hydroxyproline assay was performed using GA-fixed human pericardium according to Creemers et al. [27]. Therefore, 10 mg of dried tissue were incubated in 10 N NaOH for 1 h at 120 °C. After neutralization in 10 N HCl and centrifugation (10,000 × g, 5 min), the supernatant was diluted 1:80 and determined in duplicates. After reaction with chloramine T and DMAB the extinction was measured at 570 nm (reference wavelength 655 nm). Due to the homogeneity of the bovine pericardial tissue two samples of each bovine pericardium were implemented.

### Histological staining and quantification

Pericardial specimen were further fixed in 4% buffered formaldehyde solution for histological processing and embedded in paraffin. Sectioning was performed and slices of 3 μm thickness were transferred to specimen slides. Samples were stained by a standard hematoxylin and eosin (HE) protocol to evaluate fiber distribution, cellular content but especially to visualize vessel content. Picrosiriusred and elastica staining were performed according to the standard protocols to calculate collagen and elastin fiber positivity in histological sections of the pericardia, respectively. Samples were digitalized via slide scanner (Axio Scan.Z1, Carl Zeiss Microscopy GmbH, Jena, Germany). Collagen fiber positive area in picrosirius red stained sections, and elastica positive stained elastic fibers were quantified with Fiji software using the color deconvolution plugin and user threshold values (Supplementary Fig. 1). Picrosiriusred and elastica positive areas and vessel count were quantified in relation to total section area.

### Immunohistological staining

Mesothelial covering was detected by immunohistological staining of vimentin in human pericardia. Vimentin antibody (Merck, Darmstadt, Germany, V-2258) was diluted 1:300; isotype control was stained in parallel. Antigen retrieval was performed in citrate buffer with pH = 6. Anti-mouse Ig and DAB peroxidase substrate kits (ImmPRESS reagent, anti-mouse Ig kit and DAB kit; VectorLaboratories, California, US) were used according to the manufacturer’s instructions. Staining without primary antibody was performed as negative control.

Nuclei were co-stained by Mayer´s Hämalaun. Mesothelial covering was calculated by relating the regions with mesothelial lining to total sample surface. Lengths were measured via Fiji software and length of mesothelial lining was related to total specimen length.

### Statistical analysis

All analyses were proceeded with GraphPad Prism 9.0 and if not explained otherwise expressed as mean ± standard deviation. All data were analysed in a three-step scheme. At first, all data of one parameter (e.g., vessel count or pericardium thickness) were analysed by descriptive statistics to determine mean, standard deviation and coefficient of variation of each individuum (for both human and bovine). Shapiro-Wilk normality test was performed to test for normality. Second, the means of human vs. bovine pericardia were compared using an unpaired Student´s *t*-test, and *p* < 0.05 were set as statistically significant. If means did not differ significantly standard deviations of human vs. bovine pericardia were tested for significant differences to determine heterogeneity. If means of a parameter differed significantly coefficients of variation of human vs. bovine pericardium parameters were tested for significant differences using Student´s *t*-test. Interindividual testing of human or bovine pericardia was performed using an ordinary one-way ANOVA or Kruskal-Wallis test for each group (interindividual), followed by Tukey’s multiple comparisons or Dunn’s multiple comparisons test, respectively.

## Results

### Pericardium thickness and biomechanics

**Fig. 1:**
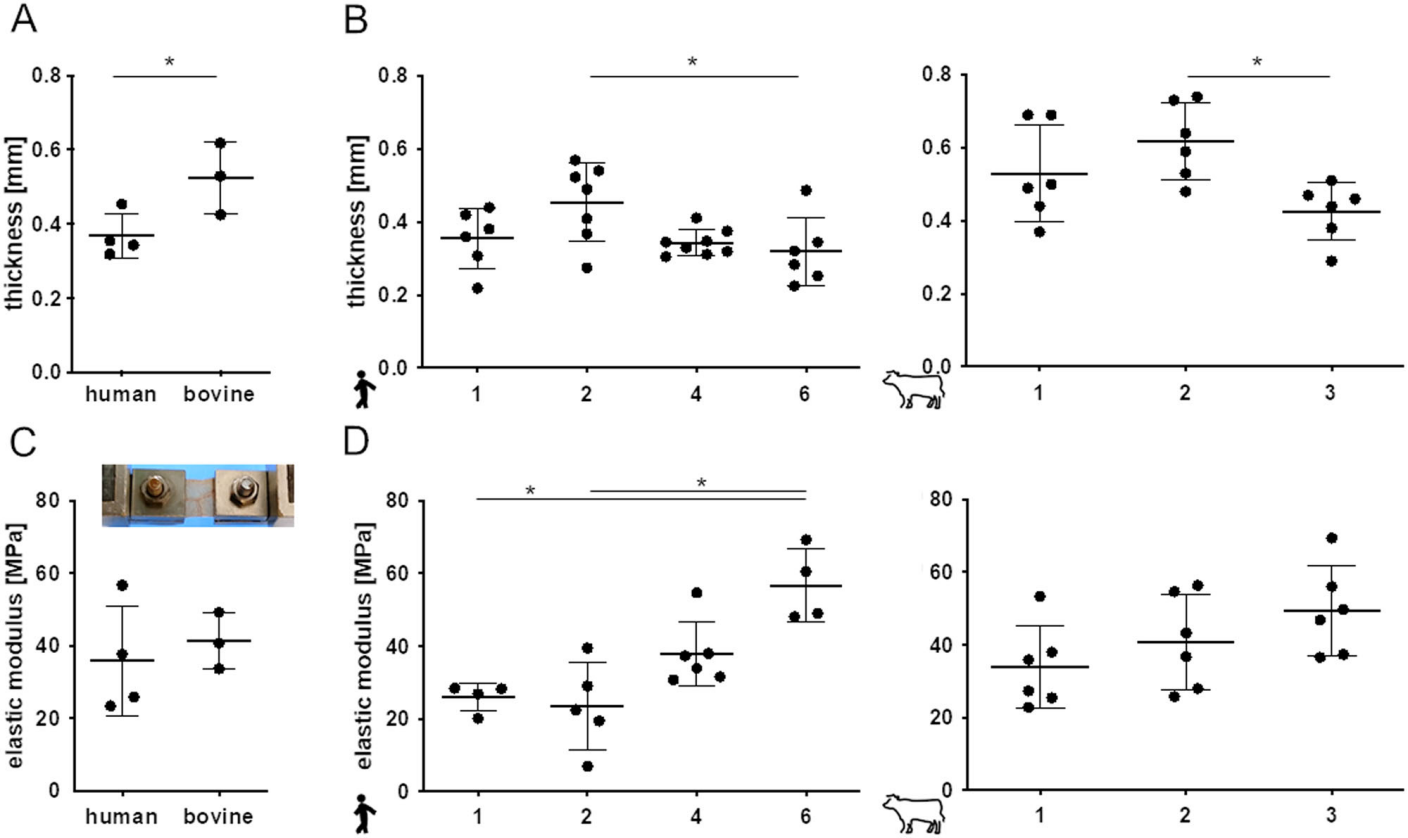
Comparison of human vs. bovine pericardium thickness and elastic moduli. Comparison of pericardia thickness **A** and interindividual differences for both species **B**. Mean elastic moduli are shown for human vs. bovine tissues **C**. No differences of elastic moduli of individual bovine pericardia **D** and interindividual variations observed comparing human samples **D**. **A**, **B**, **C** and **D**: Unpaired Student´s *t*-test and One-way ANOVA, Tukey’s test: *p < 0.5; **D** human samples: Kruskal-Wallis test, Dunn´s multiple comparison test: *p < 0.5

Thickness of human pericardia GA-fixed in the theater and equivalently treated bovine tissues were determined. Human pericardia were with a mean thickness of 0.37 ± 0.06 mm significantly thinner than bovine pericardia that exhibited a thickness of 0.52 ± 0.10 mm (Fig. 1A). In both human and bovine tissues appeared significant intra-species differences. Human pericardia sample two showed with 0.45 ± 0.11 mm the highest thickness and differed significantly from the pericardium sample six that was 0.32 ± 0.10 mm thick. Bovine pericardia samples two and three also showed with 0.62 ± 0.11 mm and 0.43 ± 0.10 mm, respectively, significant differences (Fig. 1B).

Uniaxial tensile testing of human vs. bovine pericardia revealed with 36.0 ± 15.2 MPa and 41.3 ± 7.8 MPa no significant differences of the elastic moduli evaluated (Fig. 1C). Elastic moduli in human pericardia ranged from 23.5 ± 12.0 MPa to 56.8 ± 10.1 MPa and differed significantly between samples one vs. six and two vs. six (Fig. 1D). In contrast, there was no significant difference of elastic moduli in bovine pericardia.

### Collagen content

**Fig. 2:**
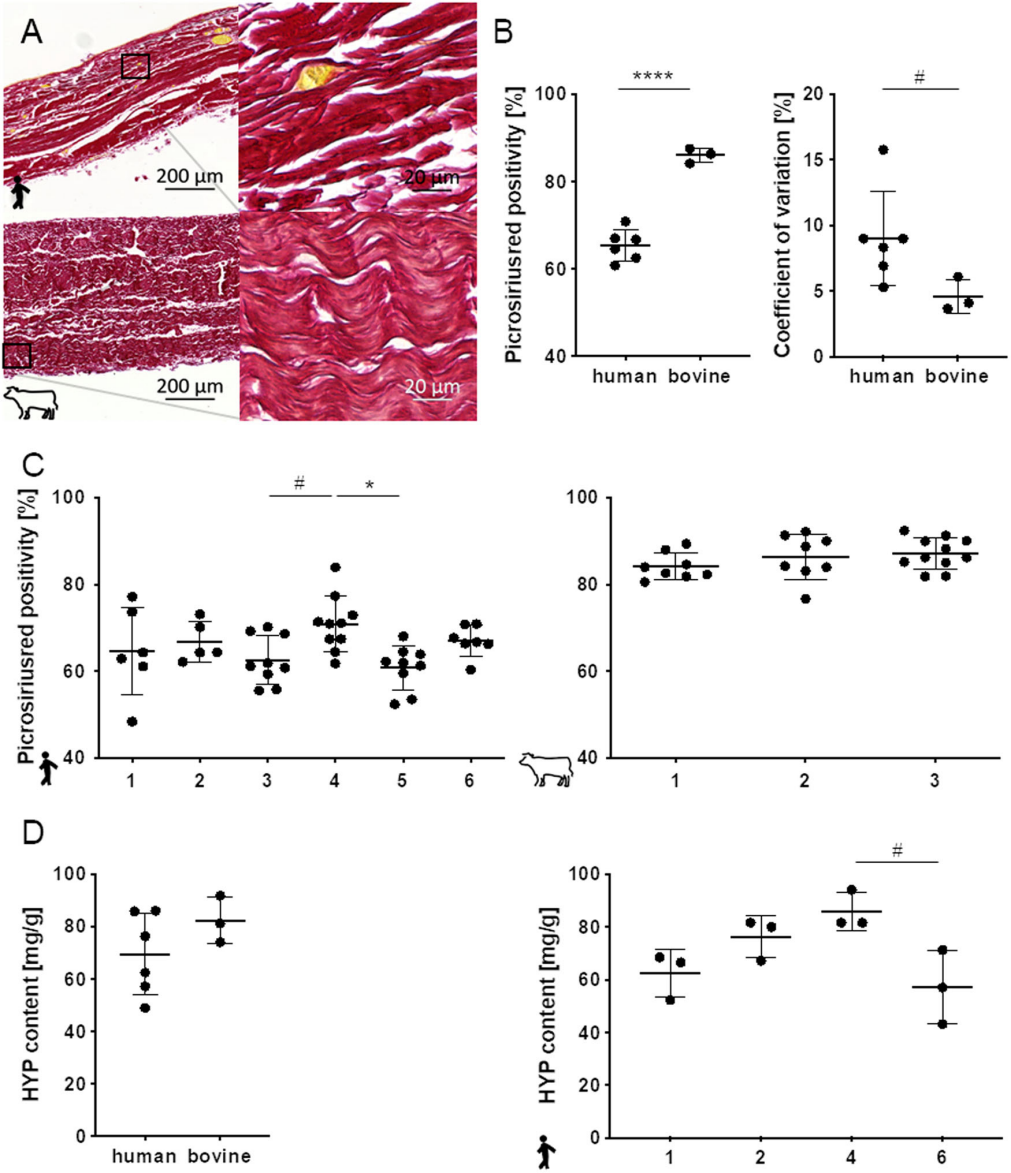
Collagen density and hydroxyproline content of human vs. bovine pericardia. **A** Comparison of distribution and wavelike structure in picrosiriusred stained bovine pericardia compared to human tissues. **B** Picrosiriusred positivity reflecting collagen distribution in the histological sections is quantified in bovine compared to human pericardium. Coefficient of variation is plotted to visualize heterogeneity of human an bovine individual pericardia **C** Interindividual comparison in picrosieriusred collagen density of single human and bovine pericardium in relation the section area. **D** Comparison of hydroxyproline levels according to pericardium weight in human vs. bovine pericardia **D** and HYP contents of individual human tissues. Unpaired Student´s *t*-test, One-way ANOVA, Tukey’s test: # 0.05 ≤ *p* < 0.1; *p < 0.5; ****p < 0.0001. **D**: Kruskal-Wallis test, Dunn´s multiple comparison test: # 0.05 ≤ *p* < 0.1

Collagen content was investigated on the one hand histologically and on the other hand by determination of hydroxyproline content related to pericardium mass. For histological evaluation sections were stained with picrosiriusred and rate of stained areas of the section was determined. Collagenous fibers appear dark red in thick, waved formations which characterize the whole pericardia sections. Human tissues exhibit strong, very densely packed bundles of fibers with reduced wave structure and larger interspaces (Fig. 2A). Bovine tissues consist of evenly distributed fibers with a uniform wavelike structure. Rate of picrosirius red positivity of the section areas in human pericardium samples was with 65.4 ± 3.6% significantly lower in human pericardia compared to bovine tissues (85.9 ± 1.5%). Coefficients of variations, although for both materials below 10%, differed with 9.0 ± 3.6% in human and with 4.6 ± 1.3% in bovine pericardia tendentially, indicating higher heterogeneity in the human tissues compared to bovine ones. Whereas bovine pericardia revealed a comparable picrosirius red positive section area rate, interindividual differences were detected for human samples four vs. five or vs. three with values of 70 ± 6.4%, 60.8 ± 5.1% and 62.5 ± 5.6%, respectively. Hydroxyproline assay revealed no significant differences in collagen content of human vs. bovine pericardia. Here the collagen content is related to tissue mass and the more densely packed fibers visible in the human tissue sections in picrosiriusred staining may be causal for the potentially controversial results. Nevertheless with 70.4 ± 13.0 mg/g dry weight the hydroxyproline content was insignificantly lower in the human tissues than in the bovine pericardium with 82.3 ± 8.9 mg/g (Fig. 2D). Interindividual differences for human pericardium were detected comparing specimen four (85.8 ± 7.2 mg/g) with sample six (57.2 ± 14.4 mg/g) with a trend (Fig. 2D). Interindividual differences in bovine material was not tested due to limited sample number in this assay. Evaluation of the standard deviations revealed a trend for higher heterogeneity in HYP content of human pericardia compared to the bovine (Supplementary Fig. 2).

### Vessel density

**Fig. 3:**
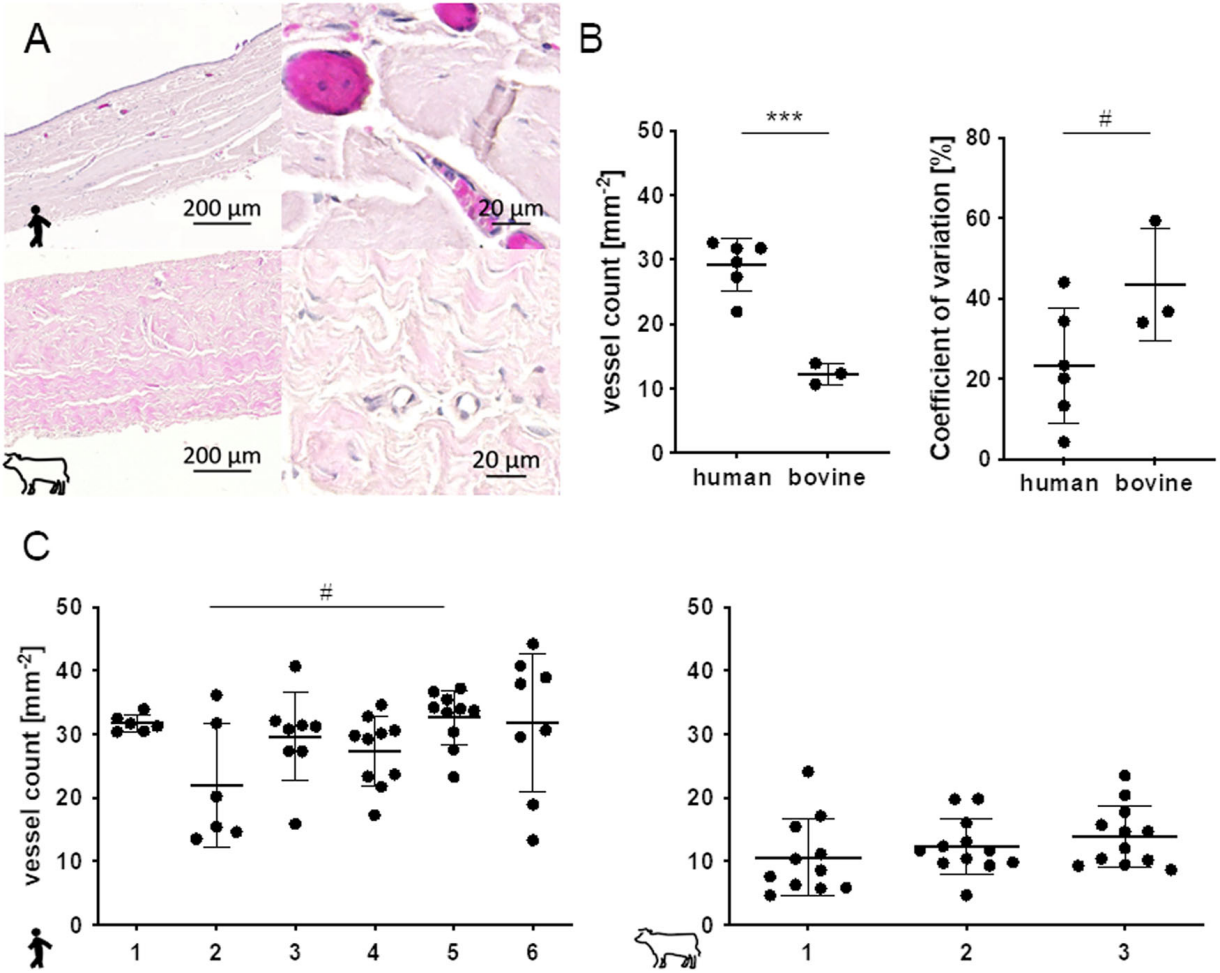
HE staining based density and heterogeneity of vessels in human vs. bovine pericardial matrix. **A** Vessels in human and bovine pericardia partially containing erythrocytes (purple). **B** Vessel count per specimen histological section area is plotted in human and bovine pericardia. **C** Differences between individual human and individual bovine pericardia samples are visualized. **B** and **C**: Unpaired Student´s *t*-test and One-way ANOVA, Tukey´s test: # 0.05 ≤ *p* < 0.1; ***p < 0.001

Pericardium vessel permeation was investigated via HE staining. A large number of vessels, equally distributed in the matrix, was found especially in the human pericardia in case of the human samples always filled with erythrocytes. In contrast the vessels visible in the bovine tissues were free from erythrocytes supposable due to more intense washing of the material before fixation (Fig. 3A). A layering of the pericardium was observed in exceptional samples shown in Supplementary Fig. 3A whereas in the human tissues no correlation of vessel location with the ECM layering was observed. In bovine pericardia that exhibit kind of layer structure the vessels were situated along the layer transitions. Human pericardia contain with 29.2 ± 4.0 mm^−2^ significantly more vessels per area than bovine tissues (12.2 ± 1.4 mm^−2^). Due to the significantly lower number of vessels in bovine pericardium with levels of only one third the coefficients of variation in the bovine material was with 43.4 ± 4.0% tendentially higher than in the human samples (23.2 ± 14.3%). The coefficients of variation in the human samples reached from 4.2 to 44%. Some samples seemed to be very homogeneous (human pericardium one) whereas others exhibited regions with very high and regions with very low numbers of vessels such as pericardium six. Interindividually there was no differences for numbers of vessels in bovine samples but with a tendency human pericardium sample five differed with 32.6 ± 4.4 mm^−2^ from sample two that showed a value of 22.0 ± 9.7 mm^−2^ (Fig. 3C).

### Content of elastic fibers

**Fig. 4:**
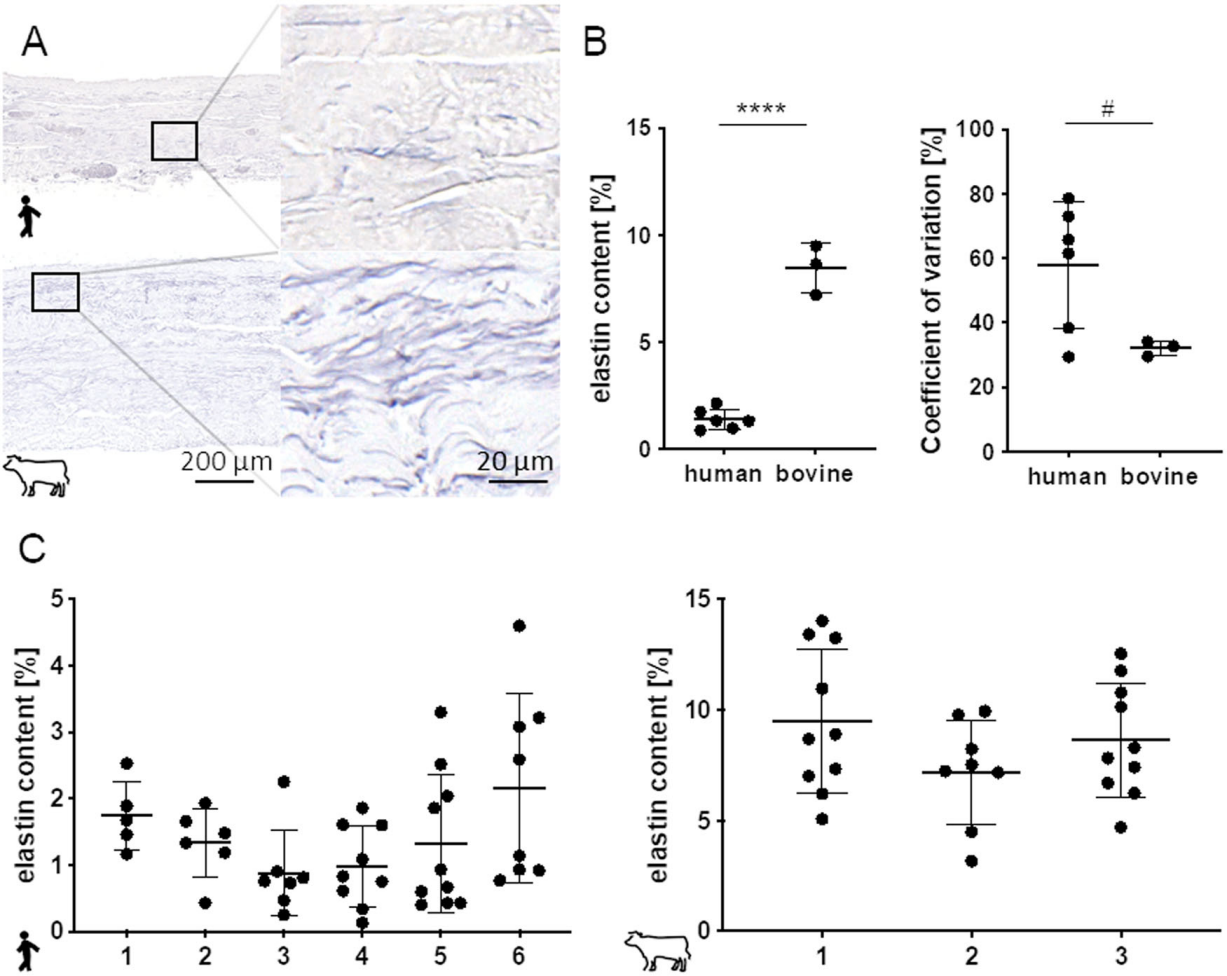
Quantification of elastin content in histological sections after elastica staining. **A** Elastic fibers embedded in the collagen matrix are visualized in dark purple. **B** Comparison of mean and coefficient of variation of human vs. bovine pericardia **C** Interindividual differences in elastin content comparing individual human pericardia or bovine tissues. **B**: Unpaired Student´s *t*-test and One-way ANOVA, Tukey´s test: # 0.05 ≤ *p* < 0.1; ****p < 0.0001

Elastin fibers beside the collagen matrix are essential for mechanobiological function of patch materials used in aortic valve replacement. Histologically via an elastin stain the content of elastin fibers, visualized in a deep purple, in human pericardia was determined and compared to bovine tissues (Fig. 4A). Elastin content was with 1.3 ± 0.5% significantly lower than in bovine samples that exhibited a six times higher value (8.5 ± 1.2%; Fig. 4B). Coefficient of variation in human specimen was tendentially higher (57.9 ± 19.6%) than in bovine pericardium (32.2 ± 2.3%) revealing a high heterogeneity in the patient material used for AVNeo. No differences were observed comparing the individual human or bovine pericardia (Fig. 4C). Elastin fiber distribution in histological sections may depend on general ECM fiber orientation in layers of the pericardium. Since only a few of the subsections showed a layering, this did not impact data acquisition. Nevertheless two examples for human and bovine pericardium are depicted in Supplementary Fig. 3B revealing the elastin fiber orientation according ECM sublayers cutting elastin fibers vertical vs. parallel to the fiber orientation.

### Mesothelial covering

**Fig. 5:**
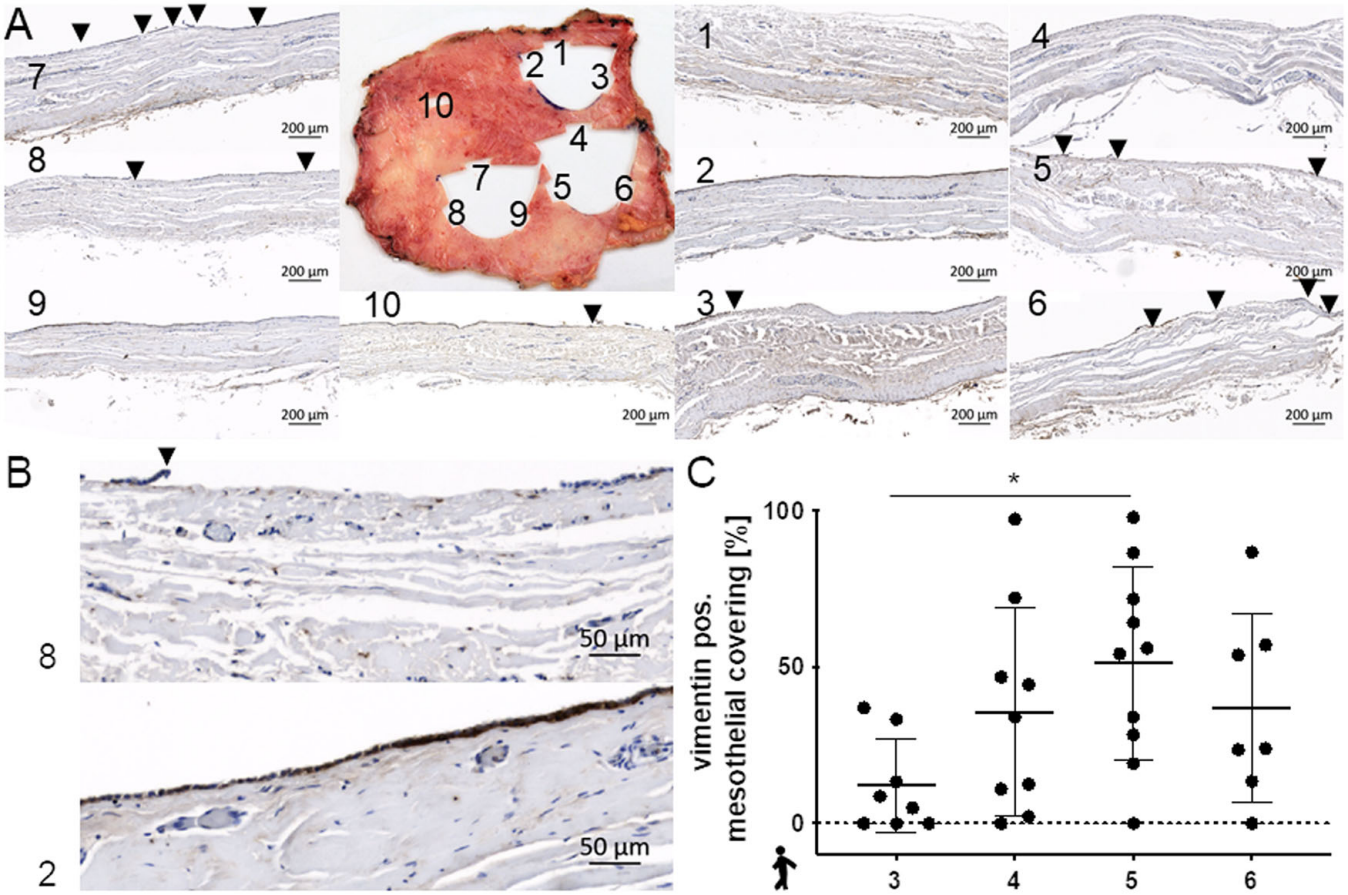
Rate and heterogeneity of mesothelial covering visualized by IHC vimentin staining; each point of mesothelial covering disruption is marked by an arrow. **A** Each sub-region of human pericardial sample 5 (1–10) is shown revealing a range from complete covering (sample 2) to absence of mesothelial cells (sample 4). Samples 2 and 8 are given enlarged in **B**. Mesothelial cell covering rate is shown comparing different areas of one human pericardium remaining after aortic valve replacement. **C**: Kruskal-Wallis test, Dunn´s multiple comparison test: *p < 0.5

Serous mesothelial covering of fibrous pericardium but also tissue orientation in implantation can impact durability of aortic valve replacement. Therefore, human pericardium samples were investigated for mesothelial lining by staining vimentin as a marker in immunohistology (Fig. 5A). Mesothelial covering within the individual patient material varied from zero percent up to 98% (Fig. 5A–C). This was reflected in individual histological specimen one to ten of patient material number five revealing samples without mesothelial lining such as number four or sample two with an intact mesothelial layer (Fig. 5A and B). Although this high variability exists, human pericardium samples three and five significantly differed in the rate of mesothelial covering with values from 12.7 ± 15 and 51.2 ± 30.7%, respectively.

## Discussion

Usage of autologous pericardium for aortic valve replacement offers immunological advantages due to absence of xenogeneic epitopes such as α-Gal responsible for degeneration of biological prostheses fabricated from bovine or porcine pericardium [17, 19, 28]. With the development of defined protocol steps according to AVNeo the utilization of patient’s pericardium was adopted more frequently [3] with excellent clinical outcomes [5]. Nevertheless patient pericardium in contrast to bovine pericardium used for bioprosthesis fabrication does not pass through material validation since explantation is performed during the surgical procedure in the theater. Aim of the presented dataset was the investigation of the human pericardium tissue heterogeneity in comparison to bovine pericardium. The purpose was to treat bovine pericardium from the corresponding tissue region as human pericardium is excised for AVNeo with the same protocol as it is performed in the theater. For the first time properties of pericardium remaining after AVNeo in clinic were compared at multiple positions adjacent to the three leaflets excised from the glutaraldehyde fixed material. Differing mechano-biological behavior, ECM composition but also tissue thickness might impact long term durability and function of the aortic valve replacement. Equivalently to Ozaki setup but with increased time of GA fixation to twenty minutes because of the higher material thickness, bovine pericardium was treated in the laboratory and was investigated as control material. Longer fixation time of twenty minutes for human pericardia has been evaluated and there was no impact on material properties [29]. It is expected that bovine pericardium processed as described herein already exhibits a higher heterogeneity than industrial produced pericardial patches that can be an additional control material in future analyses.

In general human and bovine pericardium differs in tissue thickness with significantly higher values in the bovine tissues. Nevertheless significant differences were observed between individuals of both species and the pericardium thickness does not automatically correlate to inapplicability of material. Also glutaraldehyde fixed porcine pericardium widely used for aortic valve bioprostheses [30] is much thinner than bovine tissues [31] and will be implemented in the investigation of tissue heterogeneity in ongoing studies. Depending on the annulus sizing in fabrication of bioprostheses thinner material is used for smaller diameters and comparable thickness for all three cusps is considered and adapted. Of great importance is the mechanobiological behavior of the human GA-fixed pericardium. It has been basically confirmed in comparative uniaxial tensile tests that the autologous pericardium treated with GA has sufficient tensile strength for use in aortic valve reconstruction [32]. Also in the herein presented setup uniaxial testing was performed, being aware of the assay limitations for a tissue performing its function in prominent aortic valve position with extensive multidirectional mechanical stress [28]. Elastic moduli per se did not differ between the human and bovine pericardium but comparing the individual human pericardia there was a difference resulting in concerns for long term mechanobiological strain. Values of determined elastic moduli are in the range of previously reported data using human pericardium [29]. Orientation and pericardium anisotropy may impact the experimental results but also the clinical outcome [18, 28, 29, 33–36]. Anisotropy is reported to be more pronounced in right anterior bovine pericardium and left side material is suggested for leaflet fabrication [34]. In addition to impact on leaflet mechanostability also the coaptation of the aortic valve cusp is influenced by anisotropy and collagen fiber orientation [36] especially relevant for AVNeo and application in young patients [14]. Due to the material availability in the presented setup anisotropy could not be focused in detail. Also the apex to base orientation of human pericardia was not assignable but was respected in the bovine pericardium preparation in which also the right anterior tissue was used and samples were cut longitudinally and transversally orientated in equal parts for uniaxial tensile testing. The overall bovine pericardium tissue location exerted in the presented setup (spanning the right ventricle) corresponds to regions “C-E” but also in part region “M” described in by Stiegelmeier et al. that recommend not to use the region “M” due to varying tissue thickness but also collagen fiber orientation [26]. Density of collagen fibers in the respective tissue sections referring to the excised cusps was determined by quantifying the rate of picrosiriusred positivity revealing a significant higher density of collagen in bovine material and densely packed bundles of fibers with reduced wave structure and larger interspaces in human pericardium. The more pronounced crimping and the more evenly waved structure of the bovine collagen fibers allows that the fibers are still relieved and can absorb even more tensile force [37]. Gardin et al. compared native human pericardium with samples from AVNeo and postulated that the non-waved fiber structure may be an effect upon the procedure itself, in which GA fixation of the pericardium is performed under stretch onto appropriate plates [21], an aspect that could be easily adapted in the human pericardium preparation protocol. Prestretch vs. unconstrained fixation of pericardium has also been suggested to impact pericardium anisotropy [33]. In contrast to histological quantification of collagen, hydroxy-proline content per pericardium dry weight, reflecting the collagen content did not differ between the species probable due to the more densely packed fibers in human tissue. Not only the collagen content in the bovine tissue sections was increased but also elastin fiber content was significantly higher in the bovine tissues leading to the conclusion that ECM matrix of bovine pericardium seems to be more appropriate for aortic valve replacement task. Comparing the elastin values according to the three excised cusps in one individual human pericardium a significant difference was detected (not shown) and in ongoing experimental setups this differences in tissue regions used for the individual leaflets should be focused and related to functional aspects. In contrast to the assumption that pericardium shows a layering in the samples itself this was found only in exceptional samples and could therefore not implemented in the evaluation strategy.

Despite the risk of mechanical degeneration and an overall risk of passive calcification there are more factors that might promote pericardium calcification. The vessel density in human pericardium was significantly higher than in bovine tissues. These vessels in the human matrix were mostly filled with erythrocytes that are described to be a nucleation point for calcification processes [17]. Nevertheless also recipient’s red blood cells entering vessels in bovine prosthetic tissue can impact calcification processes as well and the in general lower vessel count in bovine material might be advantageous.

Mesothelial covering of pericardium used for aortic valve replacement and orientation of the mesothelial covering towards the aorta can positively impact thrombogenicity [21, 38, 39]. Due to the minimalized preparation process and time in AVNeo a better preservation of the mesothelial cell layer than in extensive preparation procedures for bovine material can be postulated. Indeed the covering of the individual human pericardia from Ozaki preparation exhibited a great variance from zero to 98% covering comparing the individual samples but also the pericardia per se. The relevance of this parameter for longevity of aortic valve replacement and a potential protocol improvement to even better preserve the covering has to be evaluated in more detail in addition to the ability for human pericardium endothelialization in situ.

Beside the comparison of the mean values of the properties between bovine and human pericardium samples and also the means of experimental parameters between individuals of one species, the coefficient of variation was calculated and demonstrated for selected endpoints to further explore the heterogeneity. With a tendency and although the mean values in human pericardium samples were lower per se, the coefficient of variation was lower for histologically evaluated collagen and elastin content in bovine tissues. A trend for higher coefficient of variation in the human pericardium was also detected for vessel count but may in this case also depend on the higher numbers of vessel per se in the patient material. Nevertheless these results confirm the results directly comparing human individual samples and further reveal the patient sample heterogeneity. Since pericardium homogeneity is an indicator for better suitability for aortic valve replacement regarding mechanobiology and hemodynamic flow [26] these results reveal a disadvantage of patient material although limited by the low sample number investigated.

This preliminary number of (only male) individual human pericardial samples is also the reason why a correlation to patient’s characteristics is not possible although this might be very important. The mean age of the patients (all male) was 66.5 ± 7.5 years. Since age dependency of pericardial properties has been shown for GA fixed bovine pericardium comparing not only material thickness but also elastic modulus and normalized ultimate tensile strength [40] patients age but also state of disease and the medications of the individuals may impact the suitability of tissue for aortic valve replacement. A clinical comparison of outcomes according to patient age comparing studies with differing age groups [3, 12] can provide insights but also investigation of remaining patients pericardium in a large scale is envisioned.

In addition multiple patient’s data should be implemented in this evaluation process to improve selection process for patients that profit from Ozaki approach. In case of concerns according to patient age and constitution but also according to tissue properties that are evaluable during theater procedure the adoption of bovine pericardial patch or conventional bioprostheses implantation can be considered.

## Conclusion

Human pericardium in AVNeo differ from bovine pericardia in tissue thickness, biomechanics and ECM composition and exhibit a higher heterogeneity. These features may be associated to function, calcification risk and therefore to longterm durability. Beside expected immunological advantages individual longterm outcome may critically depend on pericardium age, condition and area that is selected for cusp excision. Improvement of protocol for GA fixation and the development of easily applicable end points supporting in theater selection process for appropriate material can refine decision process. In case of concerns, bovine pericardium patches or conventional bioprostheses may be preferably used.

## Supplementary information

Supplementary Fig. 1

Supplementary Fig. 2

Supplementary Fig. 3

Supplementary Fig. legends

## Data Availability

Data available on request.

## Abbreviations

AV: aortic valve
GA: glutaraldehyde
ECM: extracellular matrix

## References

[1] Ozaki S, Kawase I, Yamashita H, Uchida S, Nozawa Y, Takatoh M, et al. A total of 404 cases of aortic valve reconstruction with glutaraldehyde-treated autologous pericardium. J Thorac Cardiovasc Surg. 2014;147:301–6. 10.1016/j.jtcvs.2012.11.012 23228404 10.1016/j.jtcvs.2012.11.012

[2] Ozaki S, Kawase I, Yamashita H, Uchida S, Takatoh M, Hagiwara S, et al. Aortic valve reconstruction using autologous pericardium for aortic stenosis. Circ J. 2015;79:1504–10. 10.1253/circj.CJ-14-1092 25818901 10.1253/circj.CJ-14-1092

[3] Ozaki S, Kawase I, Yamashita H, Uchida S, Takatoh M, Kiyohara N. Midterm outcomes after aortic valve neocuspidization with glutaraldehyde-treated autologous pericardium. J Thorac Cardiovasc Surg. 2018;155:2379–87. 10.1016/j.jtcvs.2018.01.087 29567131 10.1016/j.jtcvs.2018.01.087

[4] Gasparyan V. Gasparyan method of total autologous reconstruction of the aortic valve. Braz J Cardiovasc Surg. 2020;35:821–3. 10.21470/1678-9741-2020-0197 33118748 10.21470/1678-9741-2020-0197 PMC7598984

[5] Amabile A, Krane M, Dufendach K, Baird CW, Ganjoo N, Eckstein FS, et al. Standardized aortic valve neocuspidization for treatment of aortic valve diseases. Ann Thorac Surg. 2022;114:1108–17. 10.1016/j.athoracsur.2022.03.067 35439450 10.1016/j.athoracsur.2022.03.067

[6] Ross DN. Surgical reconstruction of the aortic valve. Lancet. 1963;1:571–4. 10.1016/s0140-6736(63)92687-4 13975079 10.1016/s0140-6736(63)92687-4

[7] Amabile A, Geirsson A, Krane M. A call to standardize nomenclature for aortic valve neocuspidization: a quest for comparable outcomes. Ann Thorac Surg. 2023;115:795–6. 10.1016/j.athoracsur.2022.04.001 35439442 10.1016/j.athoracsur.2022.04.001

[8] Krane M, Amabile A, Ziegelmuller JA, Geirsson A, Lange R. Aortic valve neocuspidization (the Ozaki procedure). Multimed Man Cardiothorac Surg, 2021. 2021. 10.1510/mmcts.2021.060. 10.1510/mmcts.2021.060 34672143

[9] Dilawar I, Putra MA, Makdinata W, Billy M, Paat RK. Autologous pericardium for adult and elderly patients undergoing aortic valve replacement: a systematic review. Cirugia Cardiovasc. 2022;29:25–30. 10.1016/j.circv.2021.07.002

[10] Benedetto U, Sinha S, Dimagli A, Dixon L, Stoica S, Cocomello L, et al. Aortic valve neocuspidization with autologous pericardium in adult patients: UK experience and meta-analytic comparison with other aortic valve substitutes. Eur J Cardiothorac Surg. 2021;60:34–46. 10.1093/ejcts/ezaa472 33517391 10.1093/ejcts/ezaa472

[11] Halees ZA, Shahid MA, Sanei AA, Sallehuddin A, Duran C. Up to 16 years follow-up of aortic valve reconstruction with pericardium: a stentless readily available cheap valve?. Eur J Cardiothorac Surg. 2005;28:200–5. 10.1016/j.ejcts.2005.04.041. 16039963 10.1016/j.ejcts.2005.04.041

[12] Krane M, Boehm J, Prinzing A, Ziegelmueller J, Holfeld J, Lange R. Excellent hemodynamic performance after aortic valve neocuspidization using autologous pericardium. Ann Thorac Surg. 2021;111:126–33. 10.1016/j.athoracsur.2020.04.108 32540439 10.1016/j.athoracsur.2020.04.108

[13] Albertini A, R E, Zucchetta F, Brega C, Mikus E, Tripodi A. Aortic valve neocuspidization with glutaraldehyde-treated autologous pericardium to avoid the prosthesis-patient mismatch of a severely obese 57-year-old patient—a case report. J Vis Surg. 2022;8:1–6.

[14] Wiggins LM, Mimic B, Issitt R, Ilic S, Bonello B, Marek J, et al. The utility of aortic valve leaflet reconstruction techniques in children and young adults. J Thorac Cardiovasc Surg. 2020;159:2369–78. 10.1016/j.jtcvs.2019.09.176 31864692 10.1016/j.jtcvs.2019.09.176

[15] Marathe SP, Chavez M, Sleeper LA, Marx G, del Nido PJ, Baird CW. Modified Ozaki procedure including annular enlargement for small aortic annuli in young patients. Ann Thorac Surg. 2020;110:1364–71. 10.1016/j.athoracsur.2020.04.025 32454012 10.1016/j.athoracsur.2020.04.025

[16] Mikami T, Uchiyama H, Maeda T, Nakashima S, Satoshi M, Taku S, et al. A case of severe aortic stenosis after aortic valve neocuspidization using autologous pericardium (Ozaki Procedure). Ann Thorac Cardiovasc Surg. 2022. 10.5761/atcs.cr.21-00269. 10.5761/atcs.cr.21-00269 PMC10587478 35321992

[17] Kostyunin AE, Yuzhalin AE, Rezvova MA, Ovcharenko EA, Glushkova TV, Kutikhin AG. Degeneration of bioprosthetic heart valves: update 2020. J Am Heart Assoc. 2020;9:e018506 10.1161/JAHA.120.018506 32954917 10.1161/JAHA.120.018506 PMC7792365

[18] Straka F, Schornik D, Masin J, Filova E, Mirejovsky T, Burdikova Z, et al. A human pericardium biopolymeric scaffold for autologous heart valve tissue engineering: cellular and extracellular matrix structure and biomechanical properties in comparison with a normal aortic heart valve. J Biomater Sci Polym Ed. 2018;29:599–34. 10.1080/09205063.2018.1429732 29338582 10.1080/09205063.2018.1429732

[19] Grebenik EA, Gafarova ER, Istranov LP, Istranova EV, Ma X, Xu J, et al. Mammalian pericardium-based bioprosthetic materials in xenotransplantation and tissue engineering. Biotechnol J. 2020;15:e1900334 10.1002/biot.201900334 32077589 10.1002/biot.201900334

[20] Jiang WJ, Cui YC, Li JH, Zhang XH, Ding HH, Lai YQ, et al. Is autologous or heterologous pericardium better for valvuloplasty? A Comparative study of calcification propensity. Tex Heart Inst J. 2015;42:202–8. 10.14503/Thij-14-4296 26175630 10.14503/THIJ-14-4296 PMC4473611

[21] Gardin C, Morciano G, Ferroni L, Mikus E, Tripodi A, Pin M, et al. Biological characterization of human autologous pericardium treated with the Ozaki procedure for aortic valve reconstruction. J Clin Med. 2021. 10. 10.3390/jcm10173954. 10.3390/jcm10173954 PMC8432048 34501402

[22] Batista RJV, Dobrianskij A, Comazzi M, Neto LTL, Rocha G, Sartori F, et al. Clinical-experience with stentless pericardial aortic monopatch for aortic-valve replacement. J Thorac Cardiovasc Surg. 1987;93:19–26. 3796028

[23] Song L, Wang X, Tao C, Xu M, Fang J, Li X, et al. Trileaflet aortic valve reconstruction using bovine pericardium. Heart Lung Circ. 2021;30:1570–7. 10.1016/j.hlc.2021.03.278 33941469 10.1016/j.hlc.2021.03.278

[24] Chan J, Basu A, Di Scenza G, Bartlett J, Fan KS, Oo S, et al. Understanding aortic valve repair through Ozaki procedure: a review of literature evidence. J Card Surg. 2022;37:5202–6. 10.1111/jocs.16846 36150152 10.1111/jocs.16846

[25] Jubouri M, Tan SZCP, Mohammed I, Bashir M. Aortic valve neocuspidization using autologous versus bovine pericardium: ozaki versus Batista. J Card Surg. 2022;37:5207–9. 10.1111/jocs.16847 36150150 10.1111/jocs.16847

[26] Stieglmeier F, Grab M, Konig F, Buch J, Hagl C, Thierfelder N. Mapping of bovine pericardium to enable a standardized acquirement of material for medical implants. J Mech Behav Biomed Mater. 2021. 118. ARTN 104432. 10.1016/j.jmbbm.2021.104432. 10.1016/j.jmbbm.2021.104432 33853036

[27] Creemers LB, Jansen DC, vanVeenReurings A, vandenBos T, Everts V. Microassay for the assessment of low levels of hydroxyproline. Biotechniques. 1997;22:656–8. 10.2144/97224bm19 9105617 10.2144/97224bm19

[28] Williams DF, Bezuidenhout D, de Villiers J, Human P, Zilla P. Long-term stability and biocompatibility of pericardial bioprosthetic heart valves. Front Cardiovasc Med. 2021;8:ARTN 728577 10.3389/fcvm.2021.728577 10.3389/fcvm.2021.728577 PMC8473620 34589529

[29] Koechlin L, Isu G, Borisov V, Robles Diaz D, Eckstein FS, Marsano A, et al. Impact on mechanical properties of 10 versus 20 min treatment of human pericardium with glutaraldehyde in OZAKI procedure. Ann Thorac Cardiovasc Surg. 2021;27:273–7. 10.5761/atcs.nm.20-00125 33536387 10.5761/atcs.nm.20-00125 PMC8374088

[30] Kueri S, Kari FA, Fuentes RA, Sievers HH, Beyersdorf F, Bothe W. The use of biological heart valves types of prosthesis, durability and complications. Dtsch Arztebl Int. 2019;116:423. 10.3238/arztebl.2019.0423 31423972 10.3238/arztebl.2019.0423 PMC6706839

[31] Walker S, Schonfelder J, Tugtekin SM, Wetzel C, Hacker MC, Schulz-Siegmund M. Stabilization and sterilization of pericardial scaffolds by ultraviolet and low-energy electron irradiation. Tissue Eng Part C Methods. 2018;24:717–29. 10.1089/ten.TEC.2018.0285 30412035 10.1089/ten.tec.2018.0285 PMC6306682

[32] Yamashita H, Ozaki S, Iwasaki K, Kawase I, Nozawa Y, Umezu M. Tensile strength of human pericardium treated with glutaraldehyde. Ann Thorac Cardiovasc Surg. 2012;18:434–7. 10.5761/atcs.oa.11.01804 22572232 10.5761/atcs.oa.11.01804

[33] Zioupos P, Barbenel JC, Fisher J. Anisotropic elasticity and strength of glutaraldehyde fixed bovine pericardium for use in pericardial bioprosthetic valves. J Biomed Mater Res. 1994;28:49–57. 10.1002/jbm.820280107 8126028 10.1002/jbm.820280107

[34] Sacks MS, Chuong CJ, More R. Collagen fiber architecture of bovine pericardium. ASAIO J. 1994;40:M632–7. 10.1097/00002480-199407000-00075 8555591 10.1097/00002480-199407000-00075

[35] D’Andrea L, Cardamone M, Bogoni F, Forzinetti E, Enei V, Valle F, et al. Anisotropic mechanical response of bovine pericardium membrane through bulge test and in-situ confocal-laser scanning. J Biomech Eng. 2023;145:Artn 031009 10.1115/1.4056398 10.1115/1.4056398 36472464

[36] Liogky A, Karavaikin P, Salamatova V. Impact of material stiffness and anisotropy on coaptation characteristics for aortic valve cusps reconstructed from pericardium. Mathematics. 2021;9:ARTN 2193 10.3390/math9182193

[37] Braga-Vilela AS, Pimentel ER, Marangoni S, Toyama MH, de Campos Vidal B. Extracellular matrix of porcine pericardium: biochemistry and collagen architecture. J Membr Biol. 2008;221:15–25. 10.1007/s00232-007-9081-5 18060343 10.1007/s00232-007-9081-5

[38] Gauvin R, Marinov G, Mehri Y, Klein J, Li B, Larouche D, et al. A comparative study of bovine and porcine pericardium to highlight their potential advantages to manufacture percutaneous cardiovascular implants. J Biomater Appl. 2013;28:552–65. 10.1177/0885328212465482 23142967 10.1177/0885328212465482

[39] Grefen L, Konig F, Grab M, Hagl C, Thierfelder N. Pericardial tissue for cardiovascular application: an in-vitro evaluation of established and advanced production processes. J Mater Sci Mater Med. 2018;29:172 10.1007/s10856-018-6186-6 30392024 10.1007/s10856-018-6186-6

[40] Sizeland KH, Wells HC, Higgins J, Cunanan CM, Kirby N, Hawley A, et al. Age dependent differences in collagen alignment of glutaraldehyde fixed bovine pericardium. Biomed Res Int. 2014;2014:189197 10.1155/2014/189197 25295250 10.1155/2014/189197 PMC4180201

