# Supplementary figures and images for: Tissue requirements for the application of aortic valve neocuspidization – appropriate pericardium properties and homogeneity?
Source: J Mater Sci Mater Med. 2024 Apr 29;35(1):26. doi: 10.1007/s10856-024-06790-2 (PMC11058761; doi:10.1007/s10856-024-06790-2)

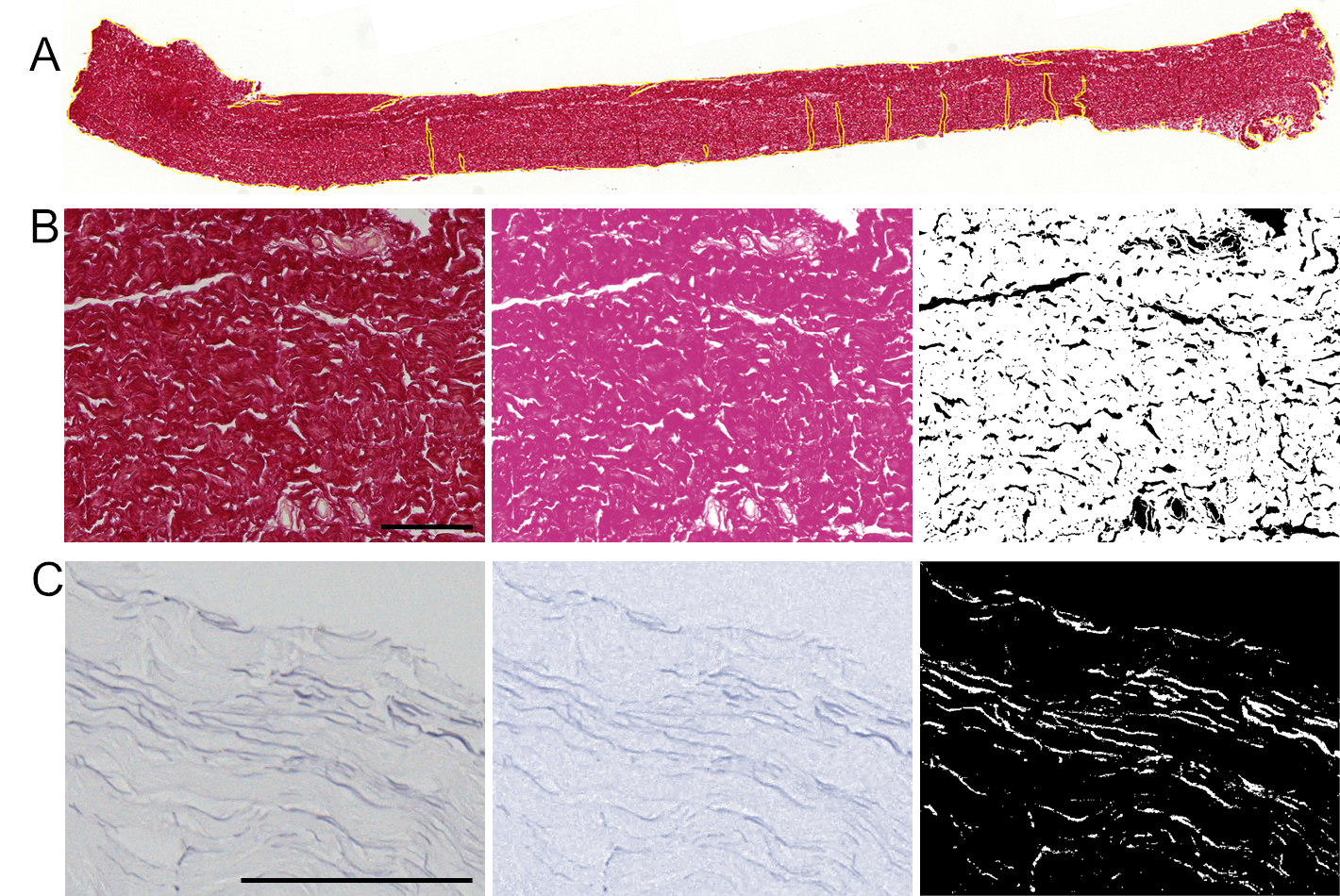

Supplement: Supplementary file 1 — Supplementary Fig. 1 [file 10856_2024_6790_MOESM1_ESM.tif]

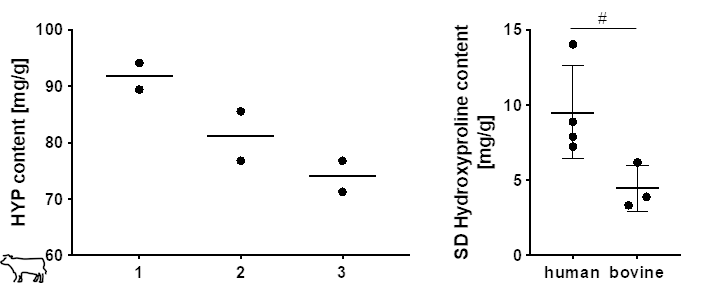

Supplement: Supplementary file 2 — Supplementary Fig. 2 [file 10856_2024_6790_MOESM2_ESM.tif]

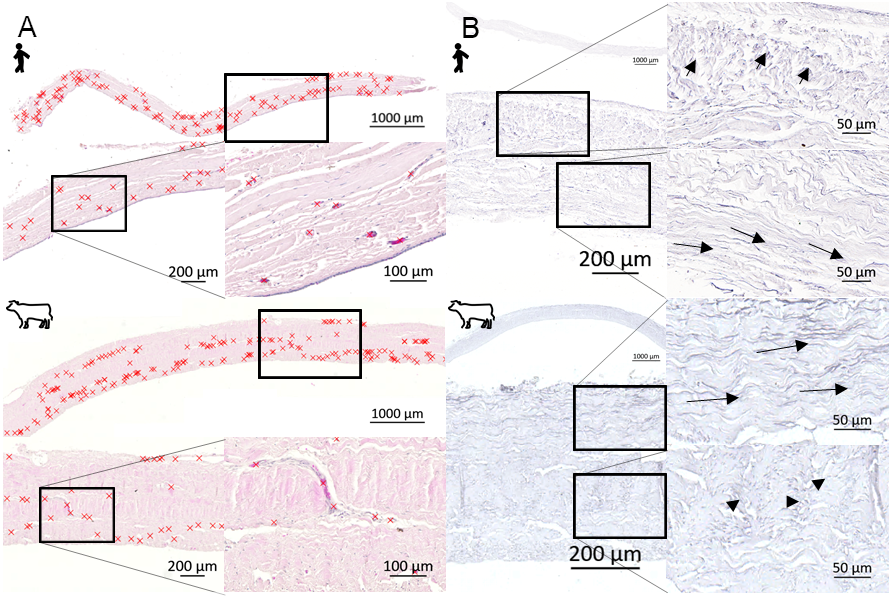

Supplement: Supplementary file 3 — Supplementary Fig. 3 [file 10856_2024_6790_MOESM3_ESM.tif]
