# Supplementary material for: Tissue requirements for the application of aortic valve neocuspidization – appropriate pericardium properties and homogeneity?
Source: J Mater Sci Mater Med. 2024 Apr 29;35(1):26. doi: 10.1007/s10856-024-06790-2 (PMC11058761; doi:10.1007/s10856-024-06790-2)
Supplement: Supplementary file 4 — Supplementary Fig. legends [file 10856_2024_6790_MOESM4_ESM.docx]

**Suppl. Fig 1** Quantification of collagen and elastin fibers based on histological tissue sections (bovine pericardium sample) stained via picrosiriusred or elastica staining. A) Determination of section area of a picrosiriusred stained bovine pericardium section using Fiji software. Overlapping tissue segments are excluded. B) Quantification of picrosiriusred positive area using colour deconvolution plugin and user specific values and thresholds (Fiji). Area of interest (white pixels in right picture) is related to the section area to determine the rate of collagen positivity. C) Quantification of the elastic fibers after elastica staining using colour deconvolution plugin and user specific values and thresholds (Fiji). Rate of elastin fiber positive area is calculated in relation to total section area. Scale bar 100 µm

**Suppl. Fig. 2** Hydroxyproline content of individual bovine pericardia (left) and comparison of interspecies standard deviation (right). Comparison of standard deviation of human vs. bovine pericardia; Unpaired t-test # 0.05 ≤p<0.1.

**Suppl. Fig. 3** Visualization of vessel distribution (HE staining) and elastin fiber (elastica staining) orientation according to tissue layering in exceptional examples that exhibit layers with different ECM orientation. A) Distribution of vessels in a human and a bovine pericardium visualized using red crosses marking each vessel cut. B) Elastin fiber orientation in different pericardial layers of human and bovine pericardium in elastica staining.
